# Association of chronic stress during studies with depressive symptoms 10 years later

**DOI:** 10.1038/s41598-025-85311-9

**Published:** 2025-01-18

**Authors:** Tobias Weinmann, Razan Wibowo, Felix Forster, Jessica Gerlich, Laura Wengenroth, Gudrun Weinmayr, Jon Genuneit, Dennis Nowak, Christian Vogelberg, Katja Radon, Britta Herbig

**Affiliations:** 1 Institute and Clinic for Occupational, Social and Environmental Medicine, LMU University Hospital, LMU Munich, Ziemssenstr. 5, 80336 Munich, Germany; 2 Institute of Epidemiology and Medical Biometry, Ulm University, Ulm, Germany; 3 Pediatric Epidemiology, Department of Pediatrics, Medical Faculty, Leipzig University, Leipzig, Germany; 4 Department of Pediatrics, Faculty of Medicine and University Hospital Carl Gustav Carus, Technische Universität Dresden, Dresden, Germany

**Keywords:** Psychological stress, Chronic stress, Mental health, Depression, Students, Depression, Psychology, Risk factors, Epidemiology

## Abstract

The long-tern implications of stress during university for individuals’ mental health are not well understood so far. Hence, we aimed to examine the potential effect of stress while studying at university on depression in later life. We analysed data from two waves of the longitudinal Study on Occupational Allergy Risks. Using the ‘work overload’ and ‘proving oneself’ scales of the Trier Inventory for Chronic Stress and the Patient Health Questionnaire-2 (PHQ-2), participants reported chronic stress during university (2007–2009, mean age 22.2 years, T1) and depressive symptoms ten years later (2017–2018, mean age 31.6 years, T2). We performed linear regression analyses to explore the association between stress during university (T1) and later depressive symptoms (T2). Participants (N = 548, 59% female) indicated rather low levels of stress and depression (PHQ-2 mean score: 1.14 (range: 0–6)). We observed evidence for a linear association between overload at T1 and depression at T2 (regression coefficient (B) = 0.270; 95% confidence interval (CI) = 0.131 to 0.409; standardised regression coefficient (β) = 0.170). Our analyses yielded evidence for an association between chronic stress while studying and risk of depressive symptoms later in life. This finding underlines the importance of implementing sustainable preventive measures against stress among students.

## Introduction

Research on stress and strain has a very long history that dates back as far as to the nineteenth century^1^. Even though stress research is a heterogeneous field with sizeable differences between studies in terms of conceptualisations and operationalisations of stress^2,3^, it has provided considerable scientific evidence for an impact of chronic stress on both physical and mental health^4,5^. For instance, chronic stress has been identified as risk factor for various adverse health outcomes such as cardiovascular disease^6^, gastrointestinal disorders^7^ and dysregulation of the immune system^8^, as well mental health issues like depression^9^ and anxiety^2^.

University students are considered especially susceptible to high levels of stress and strain with recent research suggesting that the Covid-19 pandemic has exacerbated this situation^10–14^. Consequently, students are frequently reported to be at high risk of mental health problems^15,16^, sleep disturbances^17^, problematic patterns of alcohol consumption^18,19^ and other forms of substance abuse^20,21^. For instance, a recent meta-analysis estimated the global prevalence of depression among medical students during the pandemic at 48%^22^. This may also lead to reduced psychological and physical quality of life as recently reported by a survey among students from four continents.^23^. Potential risk factors for stress and mental health disorders among students include a variety of themes including, among others, social (e.g., social support), economic (e.g., family income) and academic factors (e.g., fear of exams).^24–27^.

In Germany, the prevalence of depressive symptoms among university students during the pandemic was estimated by a meta-analysis at around 30% of the students compared to 18% before the Covid outbreak^28^, higher than the prevalence in the general German population of the same age group, which was estimated about 10% before the pandemic and 25% during the first months of the pandemic^29^. Even in the post-pandemic era, studies continue to report very high levels of depressive symptoms among university students.

For example, recent surveys among college students in China and Bangladesh both reported that even almost 50% of their participants suffered from symptoms of depression while in a study in Jordan a quarter of participants indicated severe depression^30–32^. High levels of stress and depression were also observed by a systematic review and meta-analysis on mental health in Ethiopian students. Concerning, the worldwide prevalence of psychological symptoms among medicals students, an analysis pooling data from 32 meta-analyses estimated the prevalence of depression at 32.5%^33^.

Having said that, there is also indication that levels of stress and mental health problems can recover over time, for instance after passing the entrance exams for postgraduate studies^34^. Accordingly, estimates of mental strain and disease among students may vary depending on timing of the survey, e.g., proximity to exams^35^. Furthermore, it should be noted that levels of stress and mental health impairment among non-students from the same age group may be only slightly different^36^ or even higher than among students^37^. Independently from being a student or not, the age period between 17–35 years has been identified as a particularly critical period for the development of various mental health disorders^38^.

However, most studies analysing the association between chronic stress while studying and mental health outcomes were either cross-sectional or followed students only as long as they still attended university, but not beyond. A recent scoping review about the available literature on mental health of students and graduates during the transition from university to professional life identified only twelve studies. Among these studies focusing on the university-to-work transition only few explicitly assessed mental health outcomes^39^. Consequently, whereas there is strong evidence for a relationship between stress and mental health *while* studying, the long-term implications of stress during university for individuals’ mental health in later life remain unclear.

This period of transition from student life to professional life is particularly interesting as it involves various challenges such as changes in self-esteem^40^, identity^41^ or in the surrounding ‘culture’, i.e., from university to work environments^42^, while at the same time stress-related behavioural patterns like problematic alcohol consumption may persist^43^.The period of entry into occupational life is thus potentially particularly critical for individuals’ mental health^44^. In addition, the age period (17–35 years) covering the entire cycle from entering university until transition to professional life is a peak period for the onset of various mental health disorders^38,45^. If stress during university contributes to this onset by having effects on mental health beyond graduation is not fully elucidated yet though.

Any investigation that aims to analyse the association between stress during university and individuals’ mental health after they finish their studies, i.e., enter professional life, should carefully consider the role of stress in early work life. First, occupational stress is an established risk factor for mental disorders like depression^46,47^. Secondly, during the transition from university to occupation, individuals may be particularly vulnerable to occupational stress since they are simultaneously facing various challenges as outlined in the paragraph above. Lack of social support, for example, which is a major determinant of stress at the workplace, has also been identified as an important factor during the university-to-work-transition^39^. Third, early-career employees are often reported to be disproportionally affected by unfavourable or even precarious working conditions such as fixed-term contracts that can lead to elevated stress levels and impaired health^48,49^. Ultimately, there is some indication that disadvantageous working conditions may be linked to pre-employment factors like academic performance^50^.

Based upon these considerations, we hypothesise that the potential effect of study stress on depression may be less pronounced among individuals who face lower levels of stress in their early work life. Likewise, to account for the potentially very important role of work-related stress outlined above, we suppose especially that those individuals who are not only experiencing high levels of stress while studying but also in their early work life are at particularly high risk of impaired mental health. In other terms, we hypothesise that stress in early work life may moderate the effect of stress while studying.

Based upon these considerations, the present analysis aimed to examine (1) the hypothesised effect of stress experienced during university on depressive symptoms in later life, and (2) the hypothesis of a moderating role of occupational stress.

## Methods

### Study design and population

**Fig. 1:**
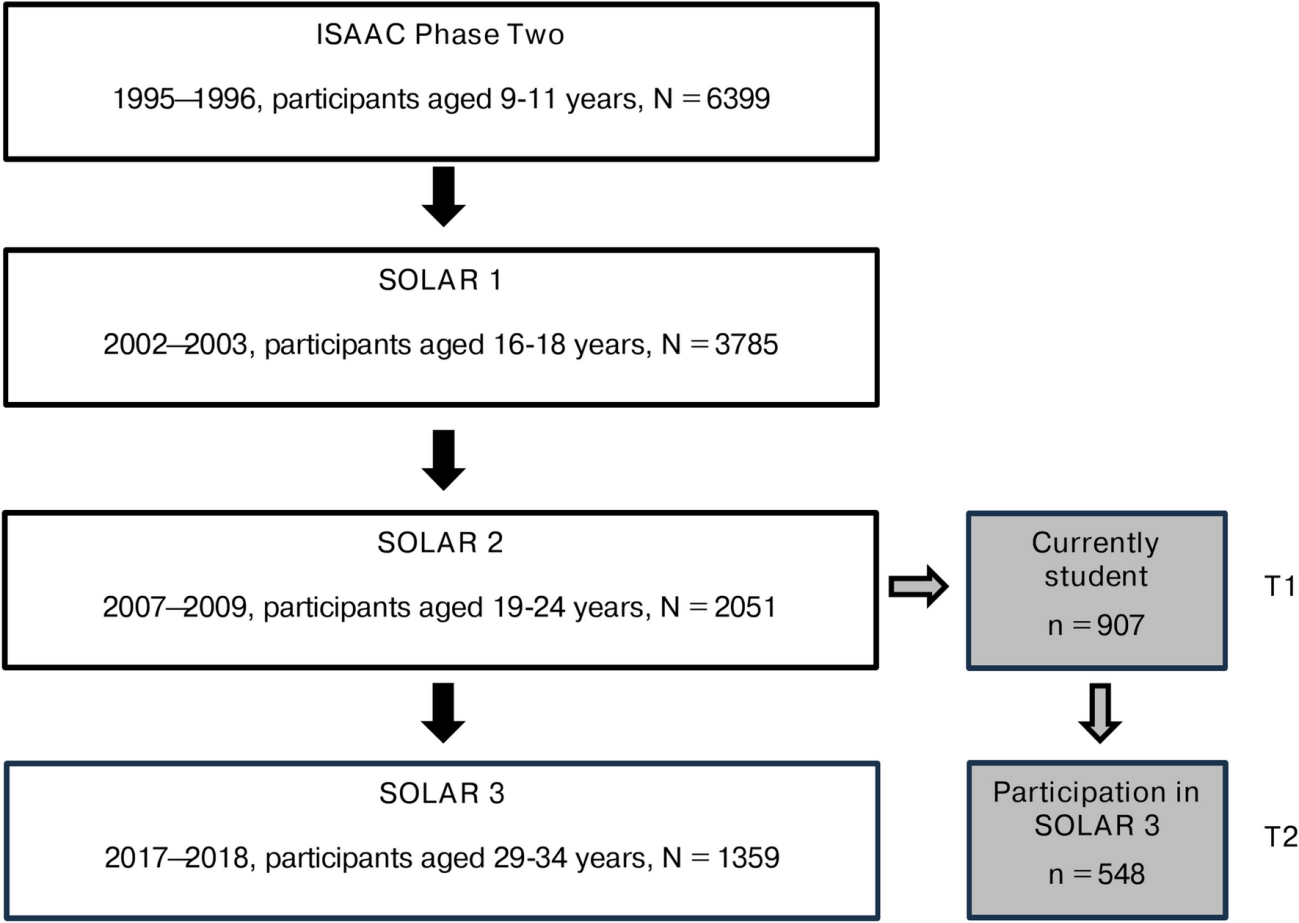
Flowchart of recruitment of study participants from ISAAC Phase Two to SOLAR 3 including period of data collection, number of participants and participants’ age range for each period of follow-up. (ISAAC, International Study of Asthma and Allergies in Childhood; SOLAR, Study on Occupational Allergy Risks).

We analysed data from two periods of the Study on Occupational Allergy Risks (SOLAR), a prospective population-based cohort study. A detailed description of the study’s rationale and methods can be found elsewhere^51,52^. Briefly, the SOLAR cohort was built upon the 6399 participants from the German study centres (Dresden and Munich) of the International Study of Asthma and Allergies in Childhood (ISAAC) Phase Two (1995–1996)^53^. In that study, eligibility criteria encompassed age group (participants had to be aged between nine and eleven years) and residency in defined geographical areas^53^. In case of the German study branch, these were the cities of Dresden und Munich. Accordingly, potential participants were randomly selected for drawn from the population registries of the cities of Dresden and Munich. SOLAR 1 was performed in 2002–2003 and included 3785 individuals who were then aged 16–18 years. Since then, they were further followed up in 2007–2009 (SOLAR 2), when they were aged 19–24 years, and in 2017–2018 (SOLAR 3, participants aged 29–34 years)^51,52^. Criteria for inclusion in SOLAR were that individuals had participated in ISAAC Phase Two and had given agreement to be re-contacted again. Furthermore, individuals had to provide written informed consent for participation in each follow-up including consent for linking data from all study phases. For the present analysis, we identified those individuals who had indicated during SOLAR 2 (T1 in the present analysis) that they were currently studying at university (N = 907). 548 of them also took part in SOLAR 3 (T2) (Fig. 1).

### Measures

All data used in this analysis were self-administered standardised questionnaire data. The main outcome depression was measured with the established and validated Patient Health Questionnaire-2 (PHQ-2)^54,55^ that screens for core symptoms of major depression over the last two weeks with two items (1) loss of interest/pleasure, (2) feeling down/depressed/hopeless) on a four-point scale from 0 (not at all) to 3 (nearly every day). For screening purposes, a cut-off point of 3 points for the summary score is recommended^56^ (sensitivity 82.9; specificity 90.0; positive predictive value 38.4 for major depressive disorder). In all analyses, we used the PHQ-2 score as a sum score. Depression was only measured in T2 (SOLAR 3).

For the assessment of the independent and moderator variables, that is, different facets of chronic stress during university (SOLAR 2, T1) and during early professional life (SOLAR 3, T2), three scales from the established Trier Inventory for the Assessment of Chronic Stress (TICS)^57,58^ were used. All items had to be rated on five-point scales indicating the frequency of experiencing the respective aspect in the last year (0 = never, 4 = very often) and were applicable to university as well as work settings. The *work overload* scale (e.g., ‘too little time to execute my daily tasks’) contained eight items and showed very good internal consistency at both measurement times (Cronbach’s α T1/T2 = 0.906/0.907). The *work discontent* scale (e.g., ‘negativity with respect to my day-to-day work’) contained four items and had good internal consistency (Cronbach’s α T1/T2 = 0.775/0.824). Finally, to assess stress specifically related to early professional life, a subscale of the TICS ‘pressure to perform’ scale was used that produced a stable independent factor in factor analysis: The *pressure to prove oneself* scale comprising six items (e.g., ‘I have tasks to do where I am under critical observation’), again with good internal consistency (Cronbach’s α T1/T2 = 0.790/0.856). In all analyses, we used the scores from the different TICS scales as mean scores.

As descriptive and potentially confounding variables the following were included: *Study programme* was not directly assessed in the questionnaire. Instead, we categorised participants into common subject classifications (see Table 3) based on the data they provided at T2 regarding their work history over the last 10 years (since time at university), e.g., a participant who reported to work as a physician at T2 was classified into the study programme ‘medicine’. This categorisation was performed independently by two authors with disagreement being reconciled by consultation of a third author and discussion until consensus was achieved. *Sex* was measured with a binary variable (female/male). *Socioeconomic status (SES)* was assessed using the educational level of one or both parents as a proxy. High SES was defined as having at least one parent with A-levels or equivalent, 12 or more years of schooling or having attended university. Low SES was assigned otherwise. *Employment status* was dichotomised into participants with formal employment relations at time of measurement (T2), that is, employees, apprentices and civil servants, and participants without formal employment, like self-employed, unemployed, maternity leave or not working because of poor health. The rationale for this categorisation was to differentiate between individuals working with/without a dependency relationship. Given the narrow age range in the SOLAR cohort, *age* was not considered as a potential confounder and is only presented for descriptive purposes. Table 1 provides an overview of all variables obtained for the present analysis.

**Table 1:** Overview of variables included in the present analysis including category, name and type of variable as well as measurement instrument.

Category	Variable name	Type of variable	Instrument
Outcome	Depression	Continuous (sum score, minimum = 0, maximum = 6)	PHQ-2
Exposure	Work overload	Continuous (mean score, minimum = 0, maximum = 4)	TICS
Exposure	Work discontent	Continuous (mean score, minimum = 0, maximum = 4)	TICS
Exposure	Pressure to prove oneself	Continuous (mean score, minimum = 0, maximum = 4)	TICS
Confounder	Sex	Binary (female, male)	Questionnaire (single item)
Confounder	Socio-economic status	Binary (low, high)	Questionnaire (derived from two items on parental education)
Confounder	Employment status	Binary (formally employed, not formally employed)	Questionnaire (single item)
Only for descriptive purposes	Age	Continuous (years)	Questionnaire (single item)
Only for descriptive purposes	Study programme	Categorical (10 categories)	Classification performed by authors based on participants’ work history

PHQ-2, Patient Health Questionnaire-2; TICS, Trier Inventory for the Assessment of Chronic Stress.

### Statistical analyses

First, we calculated descriptive statistics to provide a summary of the characteristics of the study population, including the number of missing values, absolute (n) and relative (%) numbers for categorical variables, as well as mean and standard deviation (SD) for continuous variables. For the stress and depression scales we also calculated Pearson correlation coefficients to explore relationships between these variables. Additionally, we calculated the mean scores for the three TICS scales and the PHQ-2 also by study programme including t-test for independent samples to explore differences in the mean scores between study programmes. Besides, we compared respondents and non-respondents to SOLAR 3 (T2) with respect their socio-demographic characteristics (sex, study centre, SES) and stress scores at SOLAR 2 (T1) by means of chi2 test and t-test for independent samples.

To assess the assumed association between the stress (exposure) and depression (outcome) scores, we performed hierarchical linear regression models^59^, i.e., added predictor and confounding variables to the models in blocks. Specifically, we first added the potential confounders sex (female vs. male), SES (low vs. high), and employment status (formally employed vs. not formally employed) (model 1). Next, we added the exposure variables ‘overload’ (T1) and ‘proving oneself’ (T2) (model 2) before adding an interaction term between those variables during the last step (model 3) to assess main as well as linear moderation effects. In all models, the stress and depression scores were treated as continuous variables. As effect estimates, the models provided the regression coefficient (B) and the corresponding 95% confidence interval (CI) and standard error (SE) as well as the standardised regression coefficient (β).

We selected the specific stress scales as primary exposure measures (overload for T1, proving oneself for T2) in a way that maximised the different experiences in relation to stress in different life stages. We decided not to use the ‘work discontent’ scale in our main analysis as we considered the verbalisation of the items of this scale as quite similar to the verbalisation of the two PHQ-2 items. Instead, we used the discontent scale in a sensitivity analysis.

As a sensitivity analysis, we (1) repeated the models replacing the ‘proving oneself’ score with the ‘work discontent’ score as a measure of stress during T2; (2) calculated the models separated by study programme, specifically the three programmes with the largest numbers of participants (educational science, medicine, engineering).

All analysis were performed using SPSS® version 29.0 (IBM, Armonk, USA).

## Results

### Descriptives

More than half of the participants were female and most individuals had a high SES (81%) and were in formal employment (83%) at T2 (SOLAR 3; Table 2). Concerning their chronic stress levels while they were studying (T1), participants reported a mean overload score of 1.63 (SD: 0.75) which corresponds to a very moderate average stress score (Table 3). With respect to our primary variable describing stress during early professional life (proving oneself, T2), the mean score was 2.06 (SD: 0.81). The scores for the TICS scales as well as the PHQ-2 had stronger intercorrelations at the same time point of measurement point than across the ten years between measurement points. The coefficient for the intercorrelation between overload (T1) and proving oneself (T2) was 0.139, while the coefficients for the correlation between overload (T1) and proving oneself (T2) with depression (T2) were 0.191 and 0.140, respectively (Table 3). Drop-out between T1 and T2 was slightly higher among males compared to females and among participants from the Munich study centre compared to their counterparts from Dresden (Additional file 1, Table A1).

**Table 2:** Socio-demographic characteristics of the study population by period of follow-up (SOLAR 2 and 3).

	T1 (SOLAR 2; N = 907)		T2 (SOLAR 3; N = 548)	
Variable	Mean (SD)	*Missing (n)*	Mean (SD)	*Missing (n)*
Age (years)	22.2 (0.6)	*0*	31.6	*0*

Variable	N (%)	*Missing (n)*	N (%)	*Missing (n)*
Measured before T1^a^				
Sex		*0*		*0*
Female	500 (55.1)		324 (59.1)	
Male	407 (44.9)		224 (40.9)	
Study centre		*0*		*0*
Dresden	420 (46.3)		279 (50.9)	
Munich	487 (53.7)		269 (49.1)	
Parental socio-economic status^b^		*10*		*6*
Low	167 (18.4)		97 (17.7)	
High	730 (80.5)		445 (81.2)	
Measured at T1				
Study programme		*0*		*0*
Educational science	70 (7.7)		67 (12.2)	
Medicine	50 (5.5)		39 (7.1)	
Engineering	65 (7.2)		55 (10.0)	
Natural sciences	14 (1.5)		10 (1.8)	
Humanities	43 (4.7)		36 (6.6)	
Computer science, systems & electrical engineering	42 (4.6)		34 (6.2)	
Economics & law	47 (5.2)		41 (7.5)	
Social & behavioural sciences	33 (3.6)		25 (4.6)	
Life sciences	19 (2.1)		18 (3.3)	
Unknown	524 (57.8)		223 (40.7)	
Measured at T2				
Employment status^c^		*N/A*		*3*
Formally employed	N/A		457 (83.4)	
Not formally employed	N/A		88 (16.1)	

SD, standard deviation. ^a^ Variables measure during ISAAC Phase Two / SOLAR I ^b^ Socioeconomic status defined by parental education; ^c^ Formally employed = participants with formal employment relations at time of measurement—employees, apprentices and civil servants, not formally employed = all others, e.g., students, self-employed, unemployed, maternity leave, not working because of poor health; Examples for study programmes: *Educational science*: teacher training; *Engineering*: construction engineering and architecture; *Natural sciences*: chemistry; *Humanities:* philosophy; *Computer science, systems & electrical engineering*: software engineering and programming languages; *Social & behavioural sciences*: psychology; *Life sciences:* biology, agriculture, forestry and veterinary medicine.

**Table 3:** Mean scores for stress and depression measures and their intercorrelations.

	Variables	Mean ± SD	1	2	3	4	5	6	7
1	Work overload (T1)	1.63 ± 0.75	(0.906)						
2	Work overload (T2)	1.63 ± 0.76	0.462***	(0.907)					
3	Work discontent (T1)	1.26 ± 0.69	0.355***	0.135**	(0.775)				
4	Work discontent (T2)	1.27 ± 0.75	0.176***	0.340***	0.287***	(0.824)			
5	Proving oneself (T1)	1.89 ± 0.67	0.441***	0.276***	0.080*	0.037	(0.790)		
6	Proving oneself (T2)	2.06 ± 0.81	0.139***	0.345***	– 0.023	0.025	0.316***	(0.856)	
7	Depression score (only T2)	1.14 ± 1.17	0.191***	0.422***	0.159***	0.511***	0.058	0.140***	(0.720)

Pearson correlation coefficients; Values in parentheses (diagonal): Cronbach’s Alpha; *p value ≤ 0.05, **p value ≤ 0.01, ***p value ≤ 0.001. Ranges for variables 1 to 6: mean score 0–4; depression: sum score 0–6; Sample sizes: overload and discontent T1 N = 906; proving oneself T1 N = 907; overload and discontent T2 N = 529; proving oneself T2 N = 528; depression score N = 531.

For all TICS scales, there were some slight differences between participants in different study programmes. For instance, medical students reported the highest scores on the overload scale during T1 (SOLAR 2). Even these scores, however, indicate only medium stress levels. In total, the general trend of participants reporting rather low to medium stress scores was evident across all study programmes, including those individuals that we could not allocate to a specific programme (Table 4).

**Table 4:** Summary statistics for the stress and depression scores by study programme.

	Educational science & research (a)	Medicine (b)	Engineering (c)	Natural sciences (d)	Humanities (e)	Computational science & systems (f)	Economics % law (g)	Social & behavioural sciences (h)	Life sciences (i)	Unknown (j)	*Overall difference (p*-value)
Work discontent (T1)	1.26 ± 0.65	0.96 ± 0.65^j^	1.01 ± 0.61^j^	1.15 ± 0.54	1.29 ± 0.68	1.17 ± 0.60	1.31 ± 0.79	1.44 ± 0.71	0.99 ± 0.46	1.32 ± 0.70^b,c^	< 0.001
Work discontent (T2)	1.11 ± 0.71	1.08 ± 0.54	1.19 ± 0.68	1.03 ± 0.58	1.36 ± 0.73	1.26 ± 0.94	1.31 ± 0.85	1.27 ± 0.75	1.21 ± 0.91	1.36 ± 0.75	0.252
Work overload (T1)	1.63 ± 0.74	1.89 ± 0.76^c,i^	1.43 ± 0.66^b^	1.74 ± 0.42	1.69 ± 0.59	1.46 ± 0.72	1.58 ± 0.79	1.58 ± 0.74	1.18 ± 0.65^b^	1.66 ± 0.77	0.010
Work overload (T2)	1.79 ± 0.79	1.89 ± 0.65	1.41 ± 0.74	1.58 ± 0.61	1.75 ± 0.71	1.32 ± 0.71	1.66 ± 0.93	1.76 ± 0.61	1.42 ± 0.70	1.62 ± 0.77	0.014
Prove oneself (T1)	1.89 ± 0.59	2.16 ± 0.67^c,f^	1.73 ± 0.66^b^	1.73 ± 0.49	1.89 ± 0.67	1.60 ± 0.60^b^	1.99 ± 0.64	1.78 ± 0.66	1.68 ± 0.56	1.93 ± 0.69	0.002
Prove oneself (T2)	2.16 ± 0.82	2.46 ± 0.87^c,d,f^	1.88 ± 0.68^b^	1.48 ± 0.52^b^	2.10 ± 0.82	1.75 ± 0.65^b^	2.30 ± 0.78	2.05 ± 0.89	1.87 ± 0.64	2.03 ± 0.83	< 0.001
Depression score (T2)	1.14 ± 1.32	0.86 ± 0.92	0.93 ± 1.00	0.80 ± 1.14	1.37 ± 1.17	1.21 ± 1.18	1.15 ± 1.17	1.16 ± 1.03	0.94 ± 1.35	1.22 ± 1.19	0.555

Mean ± standard deviation; T1: SOLAR 2, T2: SOLAR 3; Score range for chronic stress variables was 0 (minimum) to 4 (maximum); Score range for depression was 0 (minimum) to 6 (maximum); The superscript after the mean and standard deviation indicates that there is a statistically significant difference (p < 0.05) between particular study programmes.

**Fig. 2:**
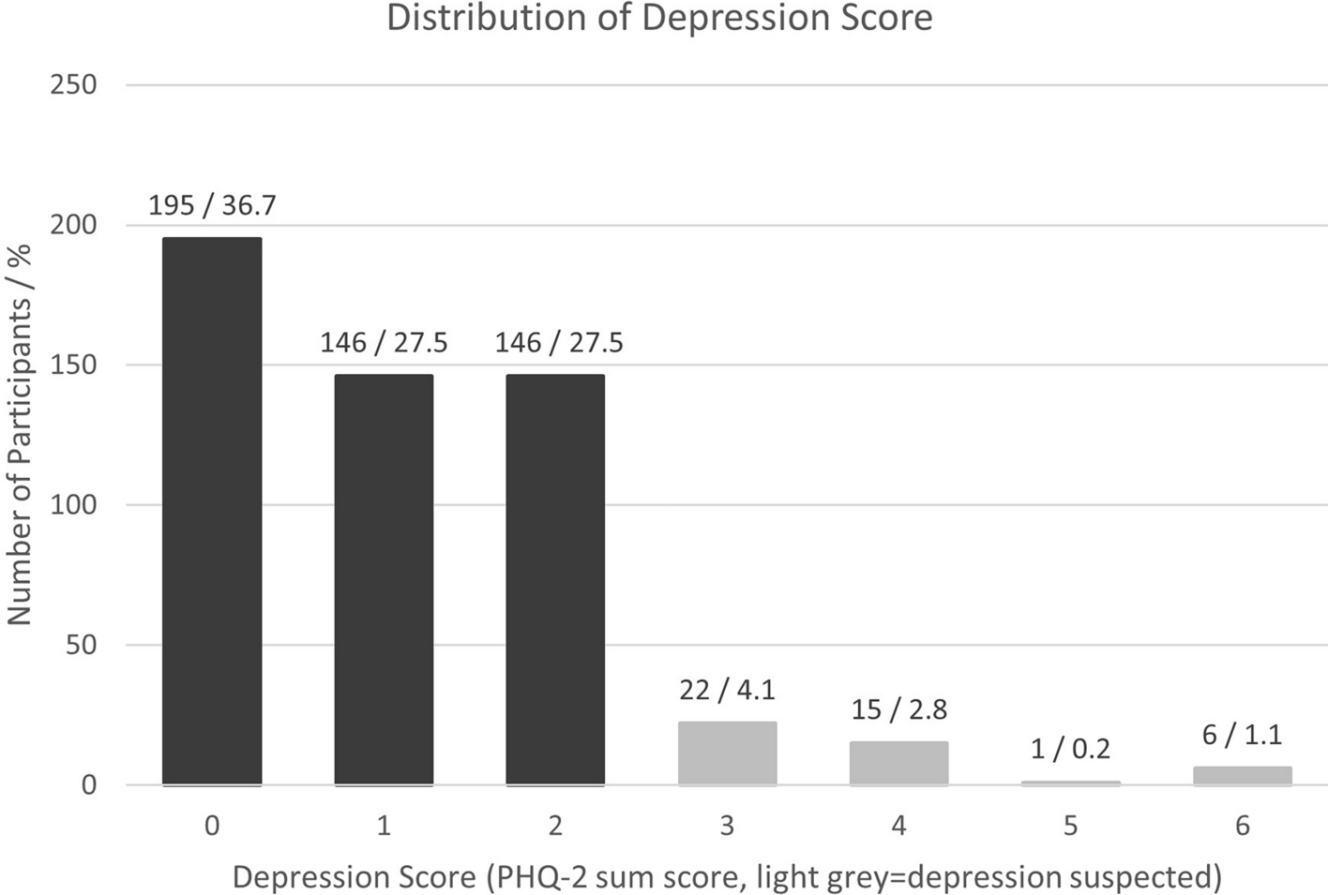
Distribution of participants´ depression score as measured by the Patient Health Questionnaire-2 (range: 0–6 points) during SOLAR 3 (T2) including absolute number and percentage of participants by sum score.

With respect to the outcome, the mean score for depressive symptoms during T2 (SOLAR 3) was 1.14 (SD: 1.17) (Table 3) with 44 participants indicating a PHQ-2 score greater or equal three points (Fig. 2). This corresponds to 8% of the participants suggesting a prevalence of major depressive disorder of about 3.1% based on the PHQ screening tools’ positive predictive value for the cut point of 3 (38.4%)^54^.

### Regression models

In the model without interaction term, we observed strong evidence for a linear association between stress (overload) at T1 and depressive symptoms at T2. Specifically, an increase in the mean overload score by one point predicted an increase in the depression sum score by approximately a quarter point (B (95% CI) = 0.270 (0.131 to 0.409); β = 0.170; p < 0.001). The mean stress score (proving oneself) at T2 was also statistically significantly associated with the depression sum score at T2 (B (95% CI) = 0.176 (0.052 to 0.300); β = 0.122; p = 0.006). The interaction effects model did not provide evidence for an interaction of overload (T1) and proving oneself (T2) in predicting depression at T2 (B (95% CI) = 0.020 (− 0.136 to 0.175); β = 0.038; p = 0.803) (Table 5).

**Table 5:** Results from the regression analysis for the association of stress (overload and proving oneself) and control variables with depression score during T2 (SOLAR 3) (N = 518).

	Model 1 (control variables)				Model 2 (linear effects)				Model 3 (interaction effect)			
	B (95% CI)	SE	β	p	B (95% CI)	SE	β	p	B (95% CI)	SE	β	p
Control variables (model 1)												
Sex	0.089 (– 0.117 to 0.296)	0.105	0.038	0.396	0.028 (– 0.177 to 0.233)	0.104	0.012	0.788	0.028 (– 0.177 to 0.232)	0.104	0.012	0.792
SES	– 0.217 (– 0.482 to 0.047)	0.135	– 0.071	0.107	– 0.229 (– 0.490 to 0.032)	0.133	-0.075	0.086	– 0.228 (– 0.491 to 0.032)	0.133	– 0.75	0.085
Employment status (T2)	0.089 (– 0.195 to 0.368)	0.143	0.027	0.545	0.132 (– 0.145 to 0.410)	0.141	0.041	0.350	0.131 (– 0.147 to 0.409)	0.141	0.040	0.355
Linear effects (model 2)												
Overload (T1)					0.270 (0.131 to 0.409)	0.071	0.170	< 0.001	0.228 (– 0.131 to 0.586)	0.182	0.144	0.213
Prove oneself (T2)					0.176 (0.052 to 0.300)	0.063	0.122	0.006	0.144 (– 0.138 to 0.426)	0.144	0.100	0.316
Interaction effect (model 3)												
Overload (T1) × Prove oneself (T2)									0.020 (– 0.136 to 0.175)	0.079	0.038	0.803
Intercept	1.196	0.191			0.402	0.242			0.470	0.365		
R^2^				0.007				0.056				0.056
ΔR				0.007				0.049*				0.000
F (df)				1.242 (3,514)				6.062 (5,512)*				5.053 (6,511)*

*p < 0.001; T1: SOLAR 2, T2: SOLAR 3; SES: socio-economic status; B: regression coefficient; CI: confidence interval; SE: standard error; β: standardised regression coefficient.

The results of the sensitivity analysis underlined these findings. When using discontent as measure for stress at T2, the linear effects model yielded strong evidence for an association of overload at T1 (B (95% CI) = 0.163 (0.040 to 0.285); β = 0.103; p = 0.009) and discontent at T2 (B (95% CI) = 0.765 (0.647 to 0.884); β = 0.489; p < 0.001) with the depression score at T2. Besides, there was no indication of an interaction effect between these two predictors (B (95% CI) = 0.061 (− 0.077 to 0.200); β = 0.099; p = 0.387) (Additional file 1, Table A2). When running separate models by study programme, the effect estimates in the linear models pointed towards the same direction like those in the models including all participants, but with a higher degree of statistical uncertainty (most likely due to the small number of participants in the stratified models). For example, among participants classified into ‘educational science’, the estimates for a linear association of overload (T1) with depressive symptoms (T2) were as follows: B (95% CI) = 0.438 (− 0.013 to 0.889); β = 0.249: p = 0.057. Furthermore, also the models stratified by study programme did not indicate an interaction effect between stress at T1 (overload) and at T2 (proving oneself) on the outcome of depressive symptoms at T2 (Additional file 1, Tables A3 to A5).

Overall, the models predicted only a rather small proportion of variance in the depression score in our study population (R^2^ = 0.056; Table 5). An exception were the models using the discontent scale (R^2^ = 0.273; Additional file 1, Table A2), but for the reasons mentioned above, we a priori decided the overload and proving oneself scales to be conceptually better suited as primary stress scales for our analyses than the discontent scale.

## Discussion

The present analysis aimed to examine the hypothesis of an association between chronic stress during university and mental health later in life. For this purpose, we assessed self-reported chronic stress among 548 German university students and their depression score ten years later. Overall, the results of our analysis confirmed the hypothesis of an association between stress while studying and risk of depression in later life even though we observed rather small effect sizes.

Furthermore, our analyses yielded no evidence that the association between stress while studying and the later risk of depression is moderated by chronic stress during early work life. Instead, we observed indication for main effects of stress during university and stress in early work life on depression. This association of work stress with the depression score is in line with the existing literature on occupational stress and mental health^46^. The lack of evidence for an interplay stress during university and stress in early work life contradicts our hypothesis that the effect of high stress while studying may be buffered by low stress in early work life. One may interpret this finding as indication that once high stress levels have initiated a process of health impairment, this process is not moderated by an increase or decrease in stress over time due to other sources, but that the crucial buffers are rather an individual’s resources (e.g., social and emotional support^60^) to cope with these stress levels^61^. Instead of moderating each other, different sources of stress may rather cumulatively contribute to allostatic load and overload^62^. Another potential explanation is that the lack of evidence for a moderation effect in our analyses is rather an issue of limited statistical power due to a not large enough sample size and low levels of stress and depression among our participants or measurement error.

To the best of our knowledge, this is one of the first studies to elucidate the long-term mental health implications of chronic stress during university by following up students over a longer period of time after their studies. It can thus be seen as a valuable extension of the existing literature on academic stress. While there is some indication of specific challenges graduates face during transition to work^39–42^, including psychological distress due to unemployment, underemployment or employment under poor psychosocial conditions^44,63^, our results imply that during this period, i.e., after finishing university, stress levels faced during university may still have an impact on individuals’ mental health. Before firm conclusion can be drawn, however, future studies need to confirm these findings.

This is especially the case as several caveats need to be kept in mind when interpreting our results. First, the SOLAR study is a population-based cohort (i.e., participants were randomly selected from the population registry when initially recruited for participation in ISAAC Phase Two), not specifically a cohort of university students. In addition, the primary outcomes of interest in SOLAR are allergic and respiratory diseases. Therefore, one may argue that not all available data were optimally suited for our research question. For example, the ‘work overload’ and ‘work discontent’ scales from the TICS were originally designed as measures rather of work-related stress, not of academic stress. Nevertheless, in our analysis the TICS scales had a good internal consistency and the verbalisation of the items of these is also applicable to university settings. As a matter of fact, we assessed all independent and dependent variables in our analysis by means of validated and commonly used instruments. The PHQ-2 for instance, while certainly not being the gold standard for a clinical diagnosis of depression, has been identified as one of the most suitable instruments for depression screening in university students by a recent literature review^64^. We therefore reckon the overall impact of information bias on our results to be rather low. Having said that, even a small degree of measurement error may have limited our ability to observe the hypothesised moderation between the two stress variables^65^. In this context, we acknowledge that one of the limitations of the PHQ-2 is its low variability compared to the PHQ-9. Therefore, we cannot rule out that some degree of measurement error occurred and that we may have seen somewhat different estimates of participants’ levels of depression had we used another tool such as the PHQ-9.

Furthermore, our study population consisted of individuals who on average had a high socio-economic status and reported rather mild stress levels. The latter might be seen as a surprise given the frequent reports of substantial stress levels among university students^66,67^ and could be interpreted as an indication of selective non-participation among individuals with higher stress levels during SOLAR 2. If so, this may limit the representativeness of our data with respect to the prevalence of chronic stress among students reducing the generalisability of our results, e.g., to other geographical areas. This assumption is underlined by the fact that the estimates for the prevalence of depression among students was lower in our study than in various other studies in other parts of the world mentioned above^30–32^. However, this should not severely impact the internal validity of our effect estimates concerning the association between stress and depression^68^. A tendency toward selection of females and participants with higher parental SES has been observed over the years of follow-up in the entire SOLAR cohort^52^, but we do not expect that this tendency has severely influenced the internal validity of the present analysis.

Concerning the outcome, we had no data on participants’ depression scores before SOLAR 3. Thus, we cannot assess the degree to which our participants already suffered from depressive symptoms during SOLAR 1 and 2, which of course may have influenced their likelihood of reporting depressive symptoms during SOLAR 3. Likewise, it is hard to judge if our results were biased by loss to follow-up in relation to the outcome, e.g., participants suffering from higher levels of depression having a higher likelihood of drop-out compared to their less mentally impaired peers, which could have hampered the internal validity of our results^69^. In general, the mean PHQ-2 scores in our study population were quite close to the average scores in a recent survey of a representative sample of German adults that also used the PHQ-2^70^.

Furthermore, it needs to be acknowledged that stress related to early professional life (measured by the ‘pressure to prove oneself’ scale) was assessed during the same time point as the outcome (depression). Therefore, we cannot preclude reverse causation between these two variables as individuals with higher depression scores may feel more stressed when performing their work compared to individuals with no or very low levels of depression. This potential inherent link between these two variables (as indicated also by the strong intercorrelation that we observed in our analysis) may be another potential explanation why we did not see an interaction between them.

In terms of uncontrolled confounding, there are various potentially relevant factors for which we had no data. For example, we could adjust our effect estimates for participants’ employment status (formally vs. not formally employed) but not for further characteristics of their current employment (e.g., fixed-term vs. permanent contract, income level) or other work-related factors like specific job demands (e.g., time pressure, emotional demands) and resources (e.g., social support) that may be associated with participants’ stress and depression levels^71,72^. Furthermore, we could not adjust our analysis for other potential stressors like critical life events^73^ or loneliness^74^. Additionally taking into account the limited amount of variance in participants’ depression scores explained by our statistical models, it appears quite likely that our results are at least partially influenced by uncontrolled confounding.

Despite those limitations, we still think that our results underline the importance of preventive measures aiming at mitigating chronic stress and mental health problems among students. In this context, there is a considerable amount of research investigating stress management and mental health interventions among university students such as cognitive behavioural therapy or mindfulness-based interventions^45,75,76^. Especially cognitive and behavioural interventions seem to have beneficial effects with respect to some outcomes like depression or anxiety disorder in students^77^. However, these types of intervention are not able to alter the underlying causes of stress among students. Instead, we consider structural interventions as a more promising approach towards sustainable improvement. For instance, studies may explore if structural interventions as successfully implemented in occupational settings^78^ could be transferred and adapted to university settings. Another structural approach may be to modify the organisation of study programmes, e.g., creating greater flexibility in the delivery of courses^79^. Even more importantly, universities should strengthen their efforts to reduce stigma around mental health as well as competition between students, increase awareness and mental health literacy among students and staff alike, foster supportive social networks and peer support, create positive learning environments and facilitate help-seeking behaviour^80–85^. A crucial element when planning, designing and implementing any form of intervention is to actively involve students right from the beginning^86^.

## Conclusions

Our analysis yielded evidence for an association of chronic stress while studying with risk of depressive symptoms later in life even though we observed rather small effect sizes. This association does not appear to be moderated by stress in early work life. Our results should be interpreted with caution though due to several potential limitations like uncontrolled confounding or bias due to loss to follow up and require confirmation by further studies. Nevertheless, they underline the importance of implementing preventive measures against stress during university. In this context, we consider structural interventions as potentially more promising and sustainable than approaches at individual level.

## Supplementary Information

Supplementary Information.

## Data Availability

The datasets presented in this article are not readily available because of data protection reasons. Requests to access the datasets should be directed to the corresponding author.

## Abbreviations

CI: Confidence interval
ISAAC: International Study of Asthma and Allergies in Childhood
PHQ-2: Patient Health Questionnaire-2
SD: Standard deviation
SES: Socio-economic status
SOLAR: Study on Occupational Allergy Risks
TICS: Trier Inventory for Chronic Stress
