# Supplementary material for: Association of chronic stress during studies with depressive symptoms 10 years later
Source: Sci Rep. 2025 Jan 18;15:2379. doi: 10.1038/s41598-025-85311-9 (PMC11742973; doi:10.1038/s41598-025-85311-9)
Supplement: Supplementary file 1 — Supplementary Information. [file 41598_2025_85311_MOESM1_ESM.docx]

**Additional file 1: Results from the sensitivity analyses**

Table A1: Comparison between respondents and non-respondents to SOLAR 3 (T2) with respect to socio-demographic characteristics and stress (TICS) scores during SOLAR 2 (T1)

|  | **Respondents (N=548)** | | **Non-respondents (N=359)** | |  |
| --- | --- | --- | --- | --- | --- |
| **Variable** | **N (%)** | ***Missing (n)*** | **N (%)** | ***Missing (n)*** | **P-Value** |
| **Sex** | | *0* |  | *0* | 0.003^a^ |
| Female | 324 (59.1) |  | 176 (49.0) |  |  |
| Male | 224 (40.9) |  | 183 (51.0) |  |  |
| **Study centre** | | *0* |  | *0* | <0.001^a^ |
| Dresden | 279 (50.9) |  | 141 (39.3) |  |  |
| Munich | 269 (49.1) |  | 218 (60.7) |  |  |
| **Parental socio-economic status^c^** | | *10* |  | *4* | 0.493^a^ |
| Low | 97 (17.7) |  | 70 (19.7) |  |  |
| High | 445 (81.2) |  | 285 (80.3) |  |  |
| **TICS scale (T1)** | **Mean ± SD** | ***Missing (n)*** | **Mean ± SD** | ***Missing (n)*** |  |
| Work overload | 1.64 ± 0.74 | *1* | 1.63 ± 0.77 | *0* | 0.903^b^ |
| Work discontent | 1.22 ± 0.66 | *1* | 1.32 ± 0.73 | *0* | 0.020^b^ |
| Proving oneself | 1.88 ± 0.67 | *0* | 1.92 ± 0.67 | *0* | 0.329^b^ |

^a^ obtained by chi² test; ^b^ obtained by t-test for independent samples; ^c^ Socioeconomic status defined by parental education;

SD, standard deviation

Table A2: Results from the regression analysis for the association of stress (overload and discontent) and control variables with depression score during SOLAR 3 (N=518)

|  | **Model 1 (control variables)** | | | | **Model 2 (linear effects)** | | | | **Model 3 (interaction effect)** | | | |
| --- | --- | --- | --- | --- | --- | --- | --- | --- | --- | --- | --- | --- |
|  | B (95% CI) | SE | β | p | B (95% CI) | SE | β | p | B (95% CI) | SE | β | p |
| **Control variables (model 1)** | | | | | | | | | | | | |
| Sex | .087 (-.119 - .293) | .105 | .037 | .409 | .016 (-.163 - .196) | .091 | .007 | .858 | .019 (-.160 - .199) | .091 | .008 | .834 |
| SES | -.220 (-.484 - .045) | .135 | -.072 | .103 | -.120 (-.348 - .108) | .116 | -.039 | .301 | -.118 (-.346 - .110) | .116 | .-.039 | .309 |
| Formally employed | .100 (-.181 - .380) | .143 | .031 | .485 | .019 (-.223 - .262) | .124 | .006 | .875 | .008 (-.237 - .252) | .124 | .002 | .951 |
| **Linear effects (model 2)** | | | | | | | | | | | | |
| Overload (T1) |  |  |  |  | .163 (.040 - .285) | .062 | .103 | .009 | .076 (-.155 - .307) | .118 | .048 | .518 |
| Discontent (T2) |  |  |  |  | .765 (.647 - .884) | .060 | .489 | < .001 | .660 (.394 – 926) | .136 | .422 | < .001 |
| **Interaction effect (model 3)** | | | | | | | | | | | | |
| Overload (T1) X discontent (T2) |  |  |  |  |  |  |  |  | .061 (-.077 - .200) | .071 | .099 | .387 |
| Intercept | 1.187 | .190 |  |  | -.018 | .203 |  |  | .131 | .266 |  |  |
| R^2^ |  |  |  | .007 |  |  |  | .272* |  |  |  | .273 |
| ΔR^2^ |  |  |  | .007 |  |  |  | .265* |  |  |  | .001 |
| F (df) |  |  |  | 1.286 (3,515) |  |  |  | 38.37 (5,513)* |  |  |  | 32.08 (6,512)* |

**p* < .001; T1: SOLAR II, T2: SOLAR III; SES: socio-economic status; B: regression coefficient; CI: confidence interval; SE: standard error; β: standardised regression coefficient

Table A3: Results from the regression analysis for the association of stress and control variables with depression score for participants in study programme ‘Educational science’ (N=65)

|  | **Step 1 (control variables)** | | | | **Step 2 (linear effects)** | | | | **Step 3 (interaction effects)** | | | |
| --- | --- | --- | --- | --- | --- | --- | --- | --- | --- | --- | --- | --- |
|  | B (95% CI) | SE | β | p | B (95% CI) | SE | β | p | B (95% CI) | SE | β | p |
| **Control variables (step 1)** | | | | | | | | | | | | |
| Sex | -.028 (-.860 - .804) | .416 | -.009 | .947 | .033 (-.756 - .822) | .394 | .011 | .934 | .105 (-.705 - .915) | .405 | .034 | .796 |
| SES | .417 (-.542 - 1.376) | .480 | .118 | .388 | .114 (-.808 - 1.036) | .461 | .032 | .805 | .082 (-.846 - 1.010) | .464 | .023 | .860 |
| Professional status | .231 (-1.057 - 1.519) | .644 | .047 | .721 | .327 (-.921 - 1.575) | .624 | .066 | .602 | .434 (-.844 - 1.712) | .638 | .087 | .499 |
| **Linear effects (step 2)** | | | | | | | | | | | | |
| Overload (T1) |  |  |  |  | .438 (-.013 - .889) | .226 | .249 | .057 | .988 (-.416 - 2.393) | .702 | .562 | .164 |
| Prove oneself (T2) |  |  |  |  | .430 (.011 - .850) | .210 | .265 | .045 | .824 (-.216 - 1.865) | .520 | .507 | .118 |
| **Interaction effects (step 3)** | | | | | | | | | | | | |
| Overload (T1) * Prove oneself (T2) |  |  |  |  |  |  |  |  | -.228 (-.779 - .323) | .275 | -.447 | .411 |
| Intercept | .600 | .762 |  |  | -.925 | .868 |  |  | -1.964 | 1.526 |  |  |
| R^2^ |  |  |  | .014 |  |  |  | .170 |  |  |  | .056 |
| ΔR |  |  |  | .014 |  |  |  | .156 |  |  |  | .000 |
| F (df) |  |  |  | .289  (3,61) |  |  |  | 2.409  (5,59)* |  |  |  | 5.053  (6,58)* |

**p* < .05; T1: SOLAR II, T2: SOLAR III; SES: socio-economic status; B: regression coefficient; CI: confidence interval; SE: standard error; β: standardised regression coefficient

Table A4: Results from the regression analysis for the association of stress and control variables with depression score for participants in study programme ‘Medicine’ (N=50)

|  | **Step 1 (control variables)** | | | | **Step 2 (linear effects)** | | | | **Step 3 (interaction effects)** | | | |
| --- | --- | --- | --- | --- | --- | --- | --- | --- | --- | --- | --- | --- |
|  | B (95% CI) | SE | β | p | B (95% CI) | SE | β | p | B (95% CI) | SE | β | p |
| **Control variables (step 1)** | | | | | | | | | | | | |
| Sex | .617 (.002 - 1.232) | .302 | .335 | .049 | .726 (.067 - 1.385) | .323 | .394 | .032 | .750 (.083 - 1.416) | .326 | .407 | .029 |
| SES | 1.039 (-.931 - 3.009) | .968 | .186 | .291 | .736 (-1.227 - 2.700) | .963 | .132 | .450 | .351 (-1.849 -2.552) | 1.078 | .063 | .747 |
| Professional status | .128 (-.685 - .942) | .400 | .056 | .750 | .279 (-.520 - 1.078) | .392 | .121 | .482 | .470 (-.467 - 1.407) | .459 | .203 | .314 |
| **Linear effects (step 2)** | | | | | | | | | | | | |
| Overload (T1) |  |  |  |  | .170 (-.256 - .595) | .208 | .138 | .422 | -.399 (-1.890 - 1.092) | .730 | -.324 | .588 |
| Prove oneself (T2) |  |  |  |  | .381 (.023 - .740) | .176 | .364 | .038 | -.020 (-1.092 - 1.051) | .525 | -.020 | .969 |
| **Interaction effects (step 3)** | | | | | | | | | | | | |
| Overload (T1) * Prove oneself (T2) |  |  |  |  |  |  |  |  | .211 (-.319 - .741) | .259 | .593 | .422 |
| Intercept | -.617 | .941 |  |  | -1.785 | 1.067 |  |  | -.481 | 1.929 |  |  |
| R^2^ |  |  |  | .135 |  |  |  | .269 |  |  |  | .284 |
| ΔR |  |  |  | .135 |  |  |  | .133 |  |  |  | .016 |
| F (df) |  |  |  | 1.720  (3,33) |  |  |  | 2.409  (5,31) |  |  |  | 1.987  (6,30) |

T1: SOLAR II, T2: SOLAR III; SES: socio-economic status; B: regression coefficient; CI: confidence interval; SE: standard error; β: standardised regression coefficient

Table A5: Results from the regression analysis for the association of stress and control variables with depression score for participants in study programme ‘Engineering’ (N=54)

|  | **Step 1 (control variables)** | | | | **Step 2 (linear effects)** | | | | **Step 3 (interaction effects)** | | | |
| --- | --- | --- | --- | --- | --- | --- | --- | --- | --- | --- | --- | --- |
|  | B (95% CI) | SE | β | p | B (95% CI) | SE | β | p | B (95% CI) | SE | β | p |
| **Control variables (step 1)** | | | | | | | | | | | | |
| Sex | -.138 (-.823 - .547) | .341 | -.065 | .687 | -.154 (-.820 - .511) | .331 | -.072 | .643 | -.157 (-.832 - .518) | .336 | -.074 | .642 |
| SES | -.052 (-.948 - .844) | .446 | -.017 | .908 | -.279 (-1.174 - .616) | .445 | -.089 | .534 | -.277 (-1.183 - .628) | .450 | -.088 | .541 |
| Professional status | -.161 (-1.262 - .941) | .549 | -.047 | .771 | -.289 (-1.410 - .815) | .553 | -.087 | .593 | -.283 (-1.448 - .883) | .579 | -.083 | .628 |
| **Linear effects (step 2)** | | | | | | | | | | | | |
| Overload (T1) |  |  |  |  | .240 (-.234 - .715) | .236 | .147 | .314 | .314 (-1.270 - 1.898) | .787 | .192 | .692 |
| Prove oneself (T2) |  |  |  |  | .464 (.032 - .896) | .215 | .317 | .036 | .514 (-.602 - 1.631) | .555 | .352 | .359 |
| **Interaction effects (step 3)** | | | | | | | | | | | | |
| Overload (T1) * Prove oneself (T2) |  |  |  |  |  |  |  |  | -.037 (-.800 - .725) | .379 | -.058 | .922 |
| Intercept | 1.180 | .668 |  |  | .317 | .781 |  |  | .204 | 1.397 |  |  |
| R^2^ |  |  |  | .004 |  |  |  | .119 |  |  |  | .120 |
| ΔR |  |  |  | .004 |  |  |  | .115 |  |  |  | .000 |
| F (df) |  |  |  | .066  (3,50) |  |  |  | 1.302  (5,48) |  |  |  | 1.064  (6,47) |

T1: SOLAR II, T2: SOLAR III; SES: socio-economic status; B: regression coefficient; CI: confidence interval; SE: standard error; β: standardised regression coefficient
